# Passive Sensing of Health Outcomes Through Smartphones: Systematic Review of Current Solutions and Possible Limitations

**DOI:** 10.2196/12649

**Published:** 2019-08-23

**Authors:** Alina Trifan, Maryse Oliveira, José Luís Oliveira

**Affiliations:** 1 Department of Electronics, Telecommunications and Informatics University of Aveiro Aveiro Portugal; 2 Institute of Electronics and Informatics Engineering of Aveiro University of Aveiro Aveiro Portugal

**Keywords:** smartphone, mobile phone, mhealth, digital health, digital medicine, digital phenotyping, health care, mHealth, self-management, systematic review

## Abstract

**Background:**

Technological advancements, together with the decrease in both price and size of a large variety of sensors, has expanded the role and capabilities of regular mobile phones, turning them into powerful yet ubiquitous monitoring systems. At present, smartphones have the potential to continuously collect information about the users, monitor their activities and behaviors in real time, and provide them with feedback and recommendations.

**Objective:**

This systematic review aimed to identify recent scientific studies that explored the passive use of smartphones for generating health- and well-being–related outcomes. In addition, it explores users’ engagement and possible challenges in using such self-monitoring systems.

**Methods:**

A systematic review was conducted, following Preferred Reporting Items for Systematic Reviews and Meta-Analyses guidelines, to identify recent publications that explore the use of smartphones as ubiquitous health monitoring systems. We ran reproducible search queries on PubMed, IEEE Xplore, ACM Digital Library, and Scopus online databases and aimed to find answers to the following questions: (1) What is the study focus of the selected papers? (2) What smartphone sensing technologies and data are used to gather health-related input? (3) How are the developed systems validated? and (4) What are the limitations and challenges when using such sensing systems?

**Results:**

Our bibliographic research returned 7404 unique publications. Of these, 118 met the predefined inclusion criteria, which considered publication dates from 2014 onward, English language, and relevance for the topic of this review. The selected papers highlight that smartphones are already being used in multiple health-related scenarios. Of those, physical activity (29.6%; 35/118) and mental health (27.9; 33/118) are 2 of the most studied applications. Accelerometers (57.7%; 67/118) and global positioning systems (GPS; 40.6%; 48/118) are 2 of the most used sensors in smartphones for collecting data from which the health status or well-being of its users can be inferred.

**Conclusions:**

One relevant outcome of this systematic review is that although smartphones present many advantages for the passive monitoring of users’ health and well-being, there is a lack of correlation between smartphone-generated outcomes and clinical knowledge. Moreover, user engagement and motivation are not always modeled as prerequisites, which directly affects user adherence and full validation of such systems.

## Introduction

### Background

Modern mobile phones have long transcended their basic use as communication tools. At present, a smartphone is equally a digital camera, a pedometer, a fitness tracker, or a virtual assistant, among others. Smartphones are familiar, unobtrusive, and discrete devices in today’s society. Their various embedded sensors along with their high ubiquity have turned them into a valuable accessory in multiple areas of research. One such area is passive sensing or self-monitoring for either predicting or classifying health-related behaviors of smartphone users [1].

Behavioral patterns such as app usage, social interactions, and a user’s activity log or contextual information such as user’s location or Wi-Fi connectivity are just a few examples of smartphone data that can be modeled into passive indicators of a user’s health or well-being [2,3]. A smartphone’s numerous embedded sensors such as digital camera, microphone, global positioning system (GPS), accelerometer, gyroscope, Wi-Fi, Bluetooth, light and sound sensors, along with their programmable platforms, enable the passive collection of user data, thus making smartphones particularly promising self-monitoring tools.

### Objectives

This systematic review aims to overview current existing literature about the passive sensing technologies and data of smartphones used to monitor users’ health status. Passive sensing does not require any explicit user involvement but rather relies on the ubiquity of smartphones for gathering meaningful data in the background, without any biases that could be introduced by users’ categorical participation. In this review, we assess recent studies on the use of smartphones as a tool for providing passive health insights, which do not use any other kind of complementary sensing or monitoring tools. Moreover, we are interested in highlighting possible limitations or system validation concerns that have been identified in the studies included in the review.

## Methods

### Search Strategy

This systematic review follows the Preferred Reporting Items for Systematic Reviews and Meta-Analyses (PRISMA) guidelines and is registered in the PROSPERO database (identifier *CRD* 4201912447). The objective of this paper was to review the literature regarding the functionality of passive sensing of modern smartphones. As such, we focused on finding the most suitable keywords for retrieving recent studies that focus on this topic. We conducted a bibliographic search on the following Web-based databases: PubMed, IEEE Xplore, ACM Digital Library, and Scopus.

The search query used for this purpose was as follows: (smartphone OR mobile) AND (sensing OR monitoring) AND well-being AND (health OR mhealth)

This strategy retrieved 7602 publications. Papers published between January 2014 and March 2019 were included in the search. We first removed duplicate titles by an automatic script and then assessed the remaining titles for relevance for the topic. The studies that passed this first assessment were further evaluated based on their abstract. The final decision on the inclusion of a study was based on its full-text evaluation.

### Inclusion and Exclusion Criteria

The titles, authors, and publication dates of the manuscripts resulting from the search were provided in a list that was further ordered by author names. Manuscripts written by the same author group and that refer to the same methodology or application were analyzed for the sake of identifying the most recent or complete publication. Having identified one such manuscript per author group, the remaining articles written by the same author group were discarded, as they would contain similar content and thus add some redundancy to the final results of the review. Other inclusion criteria were as follows:

#### Relevance for the Chosen Topic

Study focus is passive sensing. Therefore, studies in which users have to explicitly manipulate the smartphone were not considered. Publications that considered smartphone as the sole sensing device were included.

#### Publication Date

Papers published from January 1, 2014 to April 1, 2019 were included in the review. Due to the fast evolution of smartphone technologies, what existed a few years ago may be obsolete now. Therefore, we decided to include only recent manuscripts based on current technologies.

Exclusion criteria were as follows: (1) publication language other than English; (2) use of other sensing devices or external sensors; (3) user interaction with the sensing system—this review focuses on passive sensing, where users should neither be aware of the sensing process nor willingly interact with the device for this purpose; (4) unavailability of the full text of a manuscript through the library services in our research institute; (5) out of scope for this review’s target; (6) lack of results—position papers were excluded; and reviews.

### Study Selection

**Figure 1:**
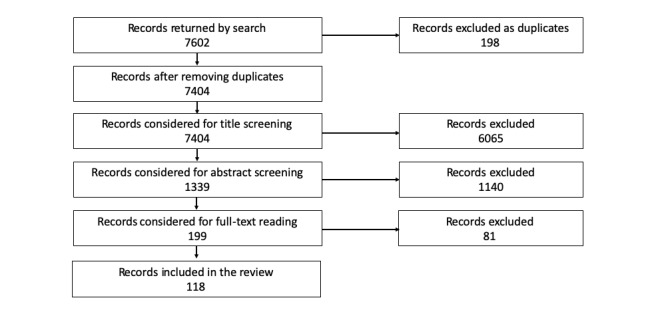
Flowchart describing the selection of the studies for the review.

On the basis of the aforementioned selection criteria, the query results were evaluated based on their titles first and then abstracts. The full text of remaining papers was read and analyzed critically to select the ones that best fulfilled the main purpose of this review. Figure 1 shows a PRISMA flow diagram [4] of the bibliographic search.

### Data Collection and Analysis

The first 2 authors of this review performed individual assessments of the papers to be included in the review. These reviewers identified possible bias in each paper, based on the Cochrane Collaboration’s risk of bias tool [5]. Finally, observations were combined into 1 spreadsheet for discussion. In case of disagreement, the third author provided advice on the final decision regarding the inclusion of a manuscript. No papers were discarded because of bias.

### Study Limitations

The search query used for the retrieval of studies for this review resulted in 7602 papers. These papers were evaluated by 2 reviewers only, which may have caused biases in the selection and screening of search results considering the topic of the review. However, when in doubt, the 2 reviewers involved the third author for an objective opinion. Another limitation of this review is the fact that all the information presented and summarized here was manually collected.

## Results

### Overview

A total of 7602 manuscripts were retrieved through the systematic search methodology described above. After removal of duplicates, we obtained 7404 studies. Of the 7404 titles inspected, 1339 were considered suitable for abstract assessment. Out of these, 199 abstracts were considered as potential candidates for the review, which led to 199 full-text retrievals and assessments. Finally, 119 manuscripts were included in the review. Table 1 shows the number of returned and selected papers from different Web-based databases.

The exclusion of a large number of papers after title assessment is because of the broadness of the search query used for study retrieval. The query was not applied to a specific field or section of a paper (eg, title or abstract), rather we looked for the terms in the query anywhere in the text of the manuscript. This led to the retrieval of a large number of papers related to the Internet of Things, smart homes, wearable monitoring systems, and robotics, as well as a considerable number of systematic reviews. The abstract evaluation further refined the number of candidate studies as, on one hand, many revealed the use of external sensors as complements of smartphones in the sensing process. On the other hand, many studies exposed explicit human interaction with the monitoring system, which would no longer satisfy 1 of the inclusion criteria for this review, passive sensing. Table 2 summarizes the percentage of excluded papers in the last step of our evaluation, based on the exclusion criteria described above.

Among the studies included for the review, we can verify that the number of published papers related to passive sensing and monitoring of health conditions using smartphones has increased over the years. More particularly, the number has doubled from 2014 to 2017 as shown in Table 3. This advocates for the research interest on the topic and strengthens the motivation of this review.

Below we provide an overview of some of the study characteristics including their main purpose, target audience, number and types of participants, and the sensing methods used. We also compiled the health conditions that have been studied and monitored using the various smartphone sensors.

**Table 1:** Number of returned and selected papers from different databases.

Studies	PubMed, n	IEEE Explore, n	ACM Digital Libraries, n	Scopus, n
Returned	2994	1604	409	2595
Included in review	44	41	10	23

**Table 2:** Distribution of rejected papers resulting from the full-text assessment.

Reason for exclusion	Excluded studies, n
Full text not available	18
Review paper	7
Off-topic	12
Preliminary work	6
User interaction required	22
Use of external sensors	17
Language (not English)	2
Same application–different study	1

**Table 3:** Number of unique returned papers by year.

Year	Studies per year, n
2014	16
2015	19
2016	30
2017	28
2018	19
2019	6

### Focus and Target Population of Included Studies

As shown in Table 3, the interest in sensing capabilities of a smartphone with the aim of improving users’ health and well-being has been increasing over the last few years. Among the selected papers for this systematic review, physical activities and mental health are 2 of the most studied health dimensions, along with sociability, students’ academic performance monitoring, and general well-being, as shown in Table 4.

Of the selected papers, 29.6% (35/118) are dedicated to the detection of users’ physical activities. Most of them aimed to recognize basic daily activities such as walking [6-22], standing or sitting [6-12,15,17-19,21-24], jogging or running [6-13,17,24], going up and down the stairs [6-10,15], lying down [10,11,15], and driving a bike [6,12,13] or vehicle [13,14,25]. In addition, 1 study tried to infer riding up and down an elevator [15], 1 assessed different activities including being stationary, limping, shuffling, and skipping [13], and 1 detected shopping and dining activities [14]. Physical activities were also explored in the sense of detecting and counting steps [26,27], distinguishing physical activity from lifestyle activities such as eating [28,29], assessing mobility in the elderly to avoid sedentary lives [30], studying its relationship with happiness including nonexercise activities [31-33], or even measuring and predicting the walking speed and distance of patients with pulmonary diseases [34].

Another health-related issue well studied in the selected papers is mental health disorders. Some of the mental health-related issues, factors, or diseases that have been investigated using smartphones are as follows: stress conditions [35-37], bipolar disorder [38-42], anxiety [42,43], schizophrenia [44,45], depression [46-49], psychotic relapse [50], mood [51-54], and affect, which have been detected, for example, using photos taken by the camera in smartphones [55,56]. A novel approach for understanding users’ emotions is the study of the typing behavior and texting speed of the users [57]. The influence of users’ exposure to natural outdoor environments on mental health has also been investigated through passive sensing [58]. Two other studies developed their monitoring solution including a recommendation system to support patients with depression to cope with their diagnosis [59,60]. Mental health systems have also been used as a tool by caregivers to access the summary of situations experienced by patients with depression [61] or to alert physicians and families if an abnormal behavior is detected in patients with mood disorders [62].

Sociability has been less studied, but it is an equally important health dimension of people’s overall well-being. It is known to have a considerable impact on the stress and anxiety levels of individuals. In fact, healthy relationships between colleagues may improve their productivity [3], united families are happier [29,63], and students cope better with their studies when surrounded by friends [64]. One way of analyzing this health dimension is by exploring interaction patterns and near locations [65,66]. An interesting approach for using the sensing capabilities of smartphones to infer the risk-taking propensity of users has been proposed in 2 studies [67,68]. One recommendation study in particular includes a feature that informs caregivers that their patients feel lonely and need additional examination [69].

Only 5.9% (7/118) of the selected papers chose to infer users’ sleep by detecting sleep patterns, irregular nights, and sleep start and end times [2,70-74]. One study focused on the correlation between sleep patterns and schizophrenia [75].

The health areas described above were investigated on an individual basis by some of the selected papers, but several other studies explored more than just 1 area to infer insights on users’ general well-being. Such systems are developed to detect the physical activities, sleep patterns, sociability levels, and location of users to either better understand and improve their behaviors or to promote awareness and self-reflection [76-80].

Self-monitoring systems have become very helpful in supporting older people with their health conditions and in the early diagnosis of abnormal conditions in the elderly. For example, the easy monitoring of cardiac parameters with smartphones, only using the users’ photographs of the finger or face, can provide a first pulse rate estimation, and users can quickly understand if something is wrong and needs additional examination [81,82]. Similarly, incidents of fall events and tremors are prone to increase in older people. Fall detection systems can quickly alert when a fall occurs, decrease the time spent on the floor, and reduce the fear of falling among the elderly [1]. On the other hand, the early diagnosis of hand tremors by passive sensing is an important contribution in the diagnosis and treatment of Parkinson disease [83-86].

Finally, 10.1% (12/118) of the selected studies developed monitoring systems specifically dedicated to students, mainly to understand how their behaviors (physical activities, sleep, and social interactions) affect their academic performance [87-89], mental health [46-48], social anxiety [43], mobility, and behaviors [18,90-93]. One study [94] presented an approach for predicting the students’ food purchase within their proximity to provide them with recommendations about healthier options.

Considering the selected papers and their described study focus, we can categorize them by disease or lifestyle monitoring. In fact, 4 studies aimed to monitor health conditions related to a specific disease, such as detecting sleep abnormalities in patients with schizophrenia or hand tremors in those with Parkinson disease, and another 12 opted to use smartphones to sense users’ daily lives to improve their general health and well-being. Among the studies that targeted a specific population, 6.9% (8/118) were on monitoring students’ lives [64,69,87,88,90,92-94] and 27.1% (33/118) on people with mental health conditions, such as depression or schizophrenia [55,60-62,75]. Senior population and workers were targeted by 3 studies each [1,3,9,30,35,69]. Among the remaining studies, 2 aimed to monitor patients with Parkinson disease [83,84], 1 targeted pulmonary patients [34], and 1 targeted family members [29]. It should be noted that among studies that aimed to monitor diseases, almost all of them targeted a specific population.

**Table 4:** Study fields of the selected papers.

Study topic	Studies per topic, n
General well-being	12
Fall detection	6
Sleep	7
Sociability	9
Mental health	33
Physical activity	35
Heart rate	2
Hand tremors and Parkinson’s	4
Respiratory issues	3
Students’ well-being	10

### Smartphone Technologies

Today’s off-the-shelf smartphones are equipped with many passive and powerful sensing technologies, which allow the continuous collection of various health-related data. Among the smartphone physical sensors, accelerometer is the most used sensor because of its low privacy and power consumption. In fact, 56.7% of the selected papers (67/118) took advantage of this sensor to gather users’ data, mostly related to physical activities.

The GPS is another commonly explored physical sensor in smartphones as it is part of most commercially available smartphones. Of the studies included, 40.6% (48/118) collected useful GPS data about users’ location and movements. This sensor was either used alone or along with Wi-Fi, Bluetooth, or accelerometer. Besides the users’ location, Bluetooth was highly used to infer levels of sociability. In fact, of the 6 papers that used Bluetooth, 4 aimed to detect users’ physical encounters.

Microphone and gyroscope are other well-studied sensors in passive systems and have been explored in 20.3% (24/118) and 16.9% (20/118) of the selected papers, respectively. A microphone is used to infer loneliness, sleep, and fall events, and a gyroscope is essentially used to detect basic physical activities.

In addition to the information collected by physical sensors, proposed solutions also collect a set of useful health-related data about the use of smartphones and users’ usage pattern. The most common ones are related to communication events including calls and text messages and smartphone usage such as screen events, light values, time spent on the phone, and device settings. Furthermore, battery level and status and app usage, used in 5.9% (7/118) of the selected papers, allow the collection of useful data about sleep.

Other health-related data can be collected from physical sensors and smartphone data, for example, camera, temporal context, and magnetometer, as shown in Table 5. The table overviews the sensors and smartphone data that are used in the selected papers.

Table 6 provides an overview of the use of smartphone sensors and data in the selected papers and different health areas.

**Table 5:** Source of the health-related data in percentage. SMS: short message service; API: application program interface; GPS: global positioning system.

Source of data	Studies, n
Camera	3
Google APIs	3
Battery level & stats	5
Magnetometer	6
Bluetooth	6
SMS & calls	13
Gyroscope	14
Microphone	17
Wi-Fi	15
Smartphone & app usage	19
GPS	48
Accelerometer	35
Others	8

**Table 6:** Summary of the smartphone sensors used in the reviewed papers.

Studied behavior	Smartphone sensors/data
General well-being	Microphone [77,95]; accelerometer [32,76,77,79,95-97]; smartphone usage [54,67,77,79]; app usage [54,78], activity recognition API^a^ [78]; text messages, calls, Wi-Fi [78,79]; GPS^b^ [67,78-80,95,97]; Bluetooth, magnetometer, gyroscope, battery level and status [79], camera [98]
Fall detection	Audio features (microphone) [1], accelerometer [99-102], GPS[99]
Sleep	App and smartphone usage [2,71,73,103]; Wi-Fi, temporal context, battery level and status [2,70,71]; accelerometer [2,71,75]; GPS, calls, text messages, activity recognition API [70]; microphone [71,74]
Sociability (loneliness, relationships)	Bluetooth [3,65,66,69]; accelerometer, gyroscope, microphone [29,104]; GPS [29,43,64,65,68,104]; Wi-Fi [29,66,69]; calls, text messages, social app usage [43,65,68,69]; emails [69]
Mental health (depression, emotions, stress level, bipolar disorder, schizophrenia)	GPS [36-39,42,44,46,47,50-52,58-61,72,105-107]; smartphone and app usage [36,39,41,42,52,53,59,60,72,106,108-110]; accelerometer [35,36,39-42,44,51,57,58,60,72,106]; cell-ID/calls [45,49,51,72,105-107]; text messages [42,45,51,55,105,107]; Wi-Fi [42,44,47,51,60]; Bluetooth [44]; microphone [36,40,44,45,51,52,62,106]; camera [55,56]; keyboard [57]; temporal context [60]; battery usage [37]; Bluetooth [37]; Google location services API, activity recognition API [61,63]
Physical activities recognition (mobility, steps counting)	Accelerometer [6-13,15-19,21-28,30,31,33,34,38,111-114]; gyroscope [6,12,15,16,18,21,22,25,27,30,33,111,113,114]; magnetometer [6,16,18,21,27,111,113]; GPS [13,14,17-20,28,115-117]; barometer [15,18,111]; gravity sensor [26], microphone [18,28]; Wi-Fi access points [17,18,28]
Heart rate measurements	Camera [81,82]
Hand tremor	Accelerometer [83,84]; gyroscope [84]
Oxygen, breath, and voice analysis	Accelerometer [118]; microphone [119,120]
Parkinson disease	GPS [86]; gyroscope [85]; accelerometer [85]
Students’ monitoring (behaviors, performance)	GPS [48,87,88,91]; microphone [87,88,90,91,93]; Wi-Fi [87,88,91,94]; accelerometer [87,90,91,93]; smartphone usage [87,90,91]; temporal context [88]; app usages, text messages, calls [48,89,90]; battery level and status [90,91]; location, weather data [92]; gyroscope, Bluetooth [91]; Google activity recognition [89]

^a^API: application programming interface.

^b^GPS: global positioning system.

One of the main advantages of the use of smartphones in health monitoring is the possibility to passively collect data. Passive data collection means that user interaction or participation is not intentional, and all sensing data come from the ubiquitous sensors of the smartphone. Of the 118 selected papers, 50 used collected data from only 1 sensor, mostly accelerometer to detect physical activities. GPS and camera were also used alone in 7 different papers. On the other hand, of the selected papers that investigated the use of several sensors, 23 used accelerometer that was essentially used along with gyroscope, GPS, Wi-Fi, and microphone to detect physical activities and general users’ behaviors.

In the spectrum of smartphone technologies, one of the main challenges that can affect the health-related collection of data when developing monitoring systems is the choice of the operating system. In fact, there are some differences and difficulties in development for Android or IOS systems, the 2 most used phone operating systems worldwide. Android is currently the most popular system and has the advantage of being convenient from the programming point of view [7]. Scanning rates of sensors are found to be superior with this operating system [3]. Furthermore, IOS hampers third-party apps to run endlessly in background, which may make the data collection difficult [91]. Of the selected papers, 56.7% (67/118) developed their system only for Android smartphones, 6 developed for both Android and IOS, and 45 did not provide any information about the chosen operating system.

### System Validation

To ensure that users of smartphone-based passive monitoring systems engage with their use requires a strong validation before releasing such systems for mass usage. Three aspects related to the validation of systems can be highlighted: the dimension of the sample of participants, the study duration, and the ground truth data that are used to compare and evaluate the results.

Validation of monitoring systems is an important phase as it can provide researchers and developers with relevant feedback and information about the accuracy and efficiency of the systems. The developed systems are tested by a sample of participants for a specific duration. Of the selected papers, about 71.1% (84/118) asked less than 50 participants to use and test their developed systems. Only few studies tested their monitoring systems with more participants: in 16.1% (19/118) of the studies, the systems were tested by 51 to 450 participants, and only 2.5% (3/118) used more than 10,000 participants in the validation phase. Although most of the papers gave information about the number of participants on their studies, 12 out of 118 (10.1%) did not provide any relevant information (see Table 7). Another aspect to be noted is that, of the papers with information about the participants, 21 out of 118 asked students to test their developed systems [2,56,64,69, 79,87,88,90-92,94]. This may be an indicator of the willingness of younger adults to engage in this area.

Study duration is also an important feature to be considered. Of the selected papers, about 20% (24 out of 118) did not provide any relevant information about the study duration. Of those studies with a specific study duration, 16.1% (19/118) lasted between 1 to 3 weeks or between 4 to 8 weeks, 12.7% (15/118) lasted between 8 to 35 weeks, and 7.6% (9/118) lasted for more than 36 weeks. Some of the papers that tried to detect physical activities chose to ask the participants to perform specific activities to test their developed systems without having a specific duration (29%, 35/118) [1,6-13,15,26,27,30,34,84] (see Table 8).

Only 1 of the selected papers did not provide any information about the number of participants and the study duration but mentioned that they had used 4 different smartphones to infer nearness based on users’ daily activities and social interactions over time and space [66].

Ground truth data allow the comparison and validation of the data collected by smartphones. Of the selected papers, about 59.3% (70/118) indicated the type of data used as ground truth, and the remaining studies did not provide any relevant information. The most used method is self-reports and questionnaires that can be performed either by a physician or provided by the participants. This method is very useful when testing monitoring systems because self-reports can be prompted to the users in their smartphones without involving any additional efforts. On the other hand, this method presents some disadvantages because the users may not always respond accurately, and results turn out to be biased. In the studies selected for this review, the questionnaire method has been essentially used to collect information about the users’ mental health [36,39,55,56,64,77,87,92], sleep [2,58,77], stress levels [35,53,55,108], and physical activities [51,77,109]. Some of the studies chose to use self-reports recognized in the health area, as for example the Patient Health Questionnaire about depression [59,60], the Pittsburgh Sleep Quality Index [75], the Unified Parkinson’s Disease Rating Scale [84], and the Beck’s Depression Inventory [80]. To collect ground truth data, dedicated devices can also be used as an alternative to questionnaires: actigraph [34], fitness devices [27,34], electrocardiogram [82], and video clips [15,30] to record participants’ physical activities. The actual pulse rate of the participants has also been collected when trying to measure cardiac parameters using the smartphone [81]. Of the selected papers, 3 asked the participants to manually label the data about them used during the study [9,65,88].

**Table 7:** Number of participants within the selected papers.

Participants, n	Studies per participant range, n
≤50	84
51–450	19
>10,000	3
Not specified	12

**Table 8:** Study duration of the selected papers.

Experiment duration, weeks	Studies, n
1 – 3	19
4 – 8	15
9 – 35	19
≥36	9
Not specified	57

### Limitations and Validation Concerns

Users’ motivations, interests, and concerns about monitoring systems may influence their adherence on using available solutions. Some of them are related to physiological aspects such as improving behaviors or monitoring health conditions such as cardiac parameters, and others are related to more technical aspects of the systems. Selected papers in this review had more concerns about technical limitations of the proposed solutions as they may affect the users’ interest and adherence to monitoring systems.

As described previously, 56.7% (67/118) papers decided to develop their systems with Android as it is simpler to develop third-party apps and because it is the most common operating system worldwide attracting more people to use the proposed systems.

Battery levels and privacy are 2 main themes approached in some of the selected papers. In fact, if these 2 aspects do not fulfill the users’ expectations, they may not use the available solutions. Of the selected papers, about 36.4% (43/118) improved the use of smartphone battery or demonstrated some concerns about its levels and hope to improve this performance in future work. The most used solution to maintain reasonable levels of battery was to decrease the sampling rates of sensors [2,13,35,70,77,91,93]. Other studies chose to pause the sampling when the battery was low [51] or to only do a unique sampling per day [65]. Finally, only 1 study [11] used accelerometer to classify activities because this sensor does not use much battery. Related to privacy, 25.4% (30/118) evidenced that privacy issues may drop users’ adherence. For example, users may want their data to be securely stored as explained and implemented in 2 studies [34,87]. Other studies chose to not store any user information on the smartphone or in the cloud [51,78], to hash all the relevant information about the user [2,3,65,78,87] or to only use the accelerometer as it raises few privacy concerns [35].

Another possible limitation of these studies is that if a developed system is tested by a sample of young adults, it may not be adapted to senior people, and results may not be accurate [1,15]. Some of the proposed models were developed and tested only with a specific population and may be too personalized, thus leading to inaccurate results when the systems are used by other populations [88,94]. Other papers pointed out the fact that personalized models produced better results than general models [2,35,70,76]. Summing up, about 16.1% (19/118) raised some concerns about the accuracy of the developed models when used on different populations. This percentage can be explained by the fact that 43.2% (51/118) of the selected papers chose to develop their systems to specific populations, and no concerns were raised by the developed models.

One of the main advantages in using a smartphone in health monitoring is its unobtrusiveness. However, almost half the selected papers required the smartphones to be on a specific body position, such as in the pocket trouser, in the handbag, or in the hand. Other studies required the smartphones to be placed in the users’ vicinity [1,2,59] or to keep it always on to make sure that the system works correctly [3,75]. These conditions may nullify the use of smartphones as it turns it into an obtrusive device for users.

Finally, considering that the main purpose of health monitoring systems is to improve users’ behaviors, health, and well-being, 37.2% (44/118) of the selected papers referred the importance of a recommendation and feedback system to make sure that users are aware of their behaviors to be able to improve them. In fact, such system features may lead to improvements in users’ daily lives and health when providing useful information to users, for example, improvements in subjects’ depression levels [60]. However, users are not willing to receive too many recommendations, as described in 1 study [55], and notifications should be sent to users only when necessary, for example, when symptoms are detected [83].

Table 9 presents a list of the selected papers that referred the described technical aspects that can have an impact on the users’ adherence to the systems.

**Table 9:** List of the selected papers that referred possible limitations either in the validation of the systems or in their use.

Concerns	Reference
Battery levels	[2,3,11,13,15,29,31,35,51,61,65,66,70,77-79,83,91,93]
Privacy	[2,3,34,35,51,61,65,69,70,78,79,87,91]
Developed models	[1,2,15,35,70,76,88,94]
Smartphone body position	[1-3,6-13,15,26,27,29,30,34,58,59,75,83,84]
Recommendations and feedback	[29-31,51,55,57,59-61,65,77,78,83,88,90-94]

## Discussion

### Comparison With Prior Work

The reviewed studies illustrate the potential of monitoring several health dimensions using only data collected from the smartphone to support users in improving their health and well-being. Several strategies for data collection were demonstrated for different health areas offering researchers several options to develop passive sensing solutions. We provide an overview of the limitations of such health-related monitoring systems reviewing the specific use of smartphone technologies to monitor, understand, and improve users’ well-being through several health dimensions. As far as we know, this is the first review that investigates the use of smartphone sensing technologies and data in health monitoring and discusses the limitations and concerns on using such systems.

Many reviewed papers focused on specific conditions such as mental health (bipolar disease, schizophrenia, major depressive disease, and mood disorder) [121-125], stress [126], cardiology [127], sleep [128], weight control through physical activities [129], management of chronic diseases in older adults [130], or in a more general way, health and well-being with particular representation of mental health and sleep [131], and psychological research (social interactions, activities, and mobility patterns) [132]. Regarding the technologies and devices used in the reviews, smartphone is the most commonly used [121-132], but only a few studies used it to collect data from its sensors [121,123,126,128,129]. In other cases, smartphones are used to prompt ecological momentary assessments to users [123,124,126], provide smartphone apps [122-125,128], or send some recommendations by short messaging service to the users [129]. Reviewed papers also consider wearable devices [122,123,125,128,130] and other devices and technologies such as tablets, fitness trackers, smartphone-connected devices, accessories, and desktop resources [123,127-130].

Compared with these reviewed papers, this review does not target a specific condition or a sensor. Our ambition was to identify all health-related aspects that can be monitored with a smartphone and to understand how far we are from using such systems as an alternative or a complement to standard clinical procedures.

### Current Challenges

Although the use of smartphones in health monitoring demonstrates to be a promising study field, available solutions still face some limitations that need to be overcome to make sure that users are comfortable and confident in using such systems. In fact, in some situations, monitoring systems may be perceived as uncomfortable, burdensome, and intrusive to users.

Regular users expect monitoring systems to be able to provide useful information and recommendations about their behaviors [133]. Given a health-related feedback, users are prone to improve their lifestyle and habits in relation with physical activities, well-being, sociability, and mental health [134,135].

Several technological aspects of health monitoring systems using smartphones should be taken into account. Among them, the most interesting one is the possibility to passively and continuously collect health-related data about users without changing their daily lives, thus turning smartphones into an unobtrusive and less burdensome tool compared with other health devices. In addition, smartphones are portable, cheaper, and more convenient than other devices and stay with the users throughout the day, which makes them a familiar tool to users [135]. Moreover, these passive systems can be used to share behavioral and health-related data with health professionals and peers. Recommendations, interventions, feedback, and reminders can be integrated to inform the users about their current state and eventually improve it [133,136].

Despite these advantages, users may still have some concerns about the use of smartphones in health monitoring. Nowadays, users decide very quickly on whether they are going to use a smartphone app or not; therefore, the developed systems should fully meet their expectations. The first aspect that the users normally evaluate is the design of apps. In addition, they hope that the developed system is easy to use and that they will not spend too much time to understand how it works. Concerns about the battery and privacy are also often raised. In fact, users expect that their battery level will not drop significantly given that these systems usually run in background continuously. Users may also discard apps because of privacy issues. Data collected using smartphones are private and should not be shared without permission or maliciously accessed. Generally, users accept to share their data with physicians or within a group of people with the same goal but are not comfortable with sharing it on social media sites, as an example. In addition, users are comfortable with apps using password access but are not willing to spend too much effort in creating accounts. Moreover, inconsistent or inappropriate results or advice may lead to the removal of a certain app. Still related to technical aspects, users expect that the app will not consume excessive space and memory and that it can run in background without affecting other smartphone functionalities [133,136].

Another important point is that users are willing to receive a reasonable number of notifications about their current state, mostly positive recommendations. The possibility to choose the frequency and timing of notifications is a feature that is interesting to them [133]. On the other hand, users are also interested in setting personal goals and achieving them. This shows that a challenge or gamification feature is prone to increase the users’ engagement [133,136].

Considering the described challenges and possible concerns, the developed systems referred in this systematic review still face some limitations that need to be overcome to meet users’ expectations and needs. First of all, validation of monitoring systems is one of the most important phases, and the systems should be tested with a sample of population highly representative of the target population for a sufficient period to collect enough data and produce results as accurate as possible. Among the selected papers, 71.1% (84/118) asked up to only 50 participants to test the developed system, and about 17.7% (21/118) of the selected papers tested their system for 1 to 3 weeks, which seems to be a short period to ensure reasonable results to make sure users are confident on using available solutions. In addition, some of the proposed systems developed models too personalized for specific populations, which may produce inaccurate results when using the system with other populations. Furthermore, the main advantage of using smartphones as a data collector is its unobtrusiveness. However, 43.2% (51/118) of the selected papers require users to keep the smartphone near them or use it on a specific body position such as hand, chest, or trouser pocket. Privacy and battery levels are other 2 aspects that need to be considered when developing monitoring systems and that make users more confident when using such systems. In fact, users insist on maintaining a good battery level despite the use of several smartphone sensors and expect that their data will be securely stored.

This review points out that smartphones may have the potential to collect health-related data and provide useful feedback to users about their health conditions. Despite the growing interest and ongoing maturation, monitoring systems may still need to be improved to attract a more diversified type of users and meet their expectations. Besides above-mentioned needs and concerns, more questions may be raised by the use of smartphones in health monitoring. In fact, at present, smartphones are used worldwide, but younger population are more comfortable using them. Health monitoring systems may be very useful to older populations, but smartphones may not be an easy and adaptable tool to them. In addition, these systems may attract more people with diagnosed diseases and specific goals, such as monitoring behaviors, controlling pulse rate, or improving their fitness, than to people with no specific goal in mind. Finally, a disadvantage of such systems is that when the users are familiar with them or have achieved their personal goals, they may not use the developed system anymore.

### Conclusions

In recent years, the capabilities of smartphones have made it possible to detect and monitor health-related behaviors of their users. Smartphones are easy to use, unobtrusive, familiar, and cheap compared with more traditional monitoring methods and come with many sensors that allow the continuous collection of health-related data, without directly interfering with users’ daily activities.

As demonstrated by this systematic review, the monitoring of health and well-being of users using a smartphone and its sensors is a promising field, hence the growing interest and ongoing maturation. Although there are a couple of predominant fields in which smartphone passive sensing contributes to the well-being of its users, considerable other domains remain underexplored. In addition, most studies focus on the prevailing use of some of the most common sensors, such as GPS or accelerometer, whereas only a handful of studies have so far explored user patterns in interaction with smartphones.

Smartphones have emerged as a good monitoring tool as they are unobtrusive, discrete, and omnipresent in today’s society and allow to continuously collect data about their users. Smartphones facilitate the diagnosis and treatment of some diseases as the care manager may have access to additional data sensed by them. Nevertheless, available solutions still present some limitations, such as privacy and battery issues, that have to be overcome to meet the users’ expectations. Finally, another aspect worth mentioning is that researchers and developers are focused on understanding what might motivate users to use such monitoring systems and arouse their confidence and long-term adherence.

## Abbreviations

GPS: global positioning system
PRISMA: Preferred Reporting Items for Systematic Reviews and Meta-Analyses

## References

[1] Cheffena M (2016). Fall detection using smartphone audio features. IEEE J Biomed Health Inform.

[2] Huang K, Ding X, Xu J, Guanling C, Ding W (2015). Monitoring sleep and detecting irregular nights through unconstrained smartphone sensing.

[3] Boonstra TW, Werner-Seidler A, O'Dea B, Larsen ME, Christensen H (2017). Smartphone app to investigate the relationship between social connectivity and mental health. Conf Proc IEEE Eng Med Biol Soc.

[4] Moher D, Liberati A, Tetzlaff J, Altman DG, PRISMA Group (2009). Preferred reporting items for systematic reviews and meta-analyses: the PRISMA statement. PLoS Med.

[5] Higgins JP, Altman DG, Gøtzsche PC, Jüni P, Moher D, Oxman AD, Savovic J, Schulz KF, Weeks L, Sterne JA, Cochrane Bias Methods Group, Cochrane Statistical Methods Group (2011). The Cochrane Collaboration's tool for assessing risk of bias in randomised trials. Br Med J.

[6] Shoaib M, Bosch S, Incel OD, Scholten H, Havinga PJ (2014). Fusion of smartphone motion sensors for physical activity recognition. Sensors (Basel).

[7] Wang C, Zhang W (2015). Activity recognition based on smartphone and dual-tree complex wavelet transform.

[8] Arif M, Bilal M, Kattan A, Ahamed SI (2014). Better physical activity classification using smartphone acceleration sensor. J Med Syst.

[9] Spinsante S, Angelici A, Lundström J, Espinilla M, Cleland I, Nugent C (2016). A mobile application for easy design and testing of algorithms to monitor physical activity in the workplace. Mob Inf Syst.

[10] Yang HC, Li YC, Liu ZY, Qiu J (2014). Harlib: A human activity recognition library on android.

[11] Aguiar B, Silva J, Rocha T, Carneiro S, Sousa I (2014). Monitoring physical activity and energy expenditure with smartphones.

[12] Li P, Wang Y, Tian Y, Zhou TS, Li JS (2017). An automatic user-adapted physical activity classification method using smartphones. IEEE Trans Biomed Eng.

[13] Zheng W, Yoshishara Y, Noel T, Tang D, Kubota N (2016). Energy-efficient activity recognition on smartphone.

[14] Wan N, Lin GE, Wilson G (2017). Addressing location uncertainties in GPS-based activity monitoring: a methodological framework. Trans GIS.

[15] del Rosario MB, Wang K, Wang J, Liu Y, Brodie M, Delbaere K, Lovell NH, Lord SR, Redmond SJ (2014). A comparison of activity classification in younger and older cohorts using a smartphone. Physiol Meas.

[16] Merchán-Baeza JA, González-Sánchez M, Cuesta-Vargas AI (2018). Using smartphones to collect quantitative data on lower limb functionality in people who have suffered a stroke. J Stroke Cerebrovasc Dis.

[17] Incel OD, Ozgovde A (2018). Arservice: a smartphone based crowd-sourced data collection and activity recognition framework. Procedia Comput Sci.

[18] Mafrur R, Nugraha IG, Choi D (2015). Modeling and discovering human behavior from smartphone sensing life-log data for identification purpose. Hum Cent Comput Inf Sci.

[19] Lee K, Kwan MP (2018). Physical activity classification in free-living conditions using smartphone accelerometer data and exploration of predicted results. Comput Environ Urban Syst.

[20] Solanas A, Batista E, Borras F, Martínez-Ballesté A, Patsakis C (2015). Wandering analysis with mobile phones: On the relation between randomness and wandering.

[21] Capela NA, Lemaire ED, Baddour N, Rudolf M, Goljar N, Burger H (2016). Evaluation of a smartphone human activity recognition application with able-bodied and stroke participants. J Neuroeng Rehabil.

[22] Hnoohom N, Mekruksavanich S, Jitpattanakul A (2017). Human activity recognition using triaxial acceleration data from smartphone and ensemble learning.

[23] Bort-Roig J, Puig-Ribera A, Contreras RS, Chirveches-Pérez E, Martori JC, Gilson ND, McKenna J (2018). Monitoring sedentary patterns in office employees: validity of an m-health tool (Walk@Work-App) for occupational health. Gac Sanit.

[24] Juen J, Cheng Q, Prieto-Centurion V, Krishnan JA, Schatz B (2014). Health monitors for chronic disease by gait analysis with mobile phones. Telemed J E Health.

[25] Chu H, Raman V, Shen J, Kansal A, Bahl V, Choudhury RR (2014). I am a smartphone and I know my user is driving.

[26] Tang Z, Guo Y, Chen X (2016). Self-adaptive step counting on smartphones under unrestricted stepping modes.

[27] Khedr K, El-Sheimy N (2017). A smartphone step counter using IMU and magnetometer for navigation and health monitoring applications. Sensors (Basel).

[28] Lutrek M, Cvetkovi B, Mirchevska V, Kafal O, Romero AE, Stathis K (2015). Recognising lifestyle activities of diabetic patients with a smartphone.

[29] Gu F, Niu J, He Z, Jin X (2017). Familypal: An effective system for detecting family activities based on smartphone.

[30] Madhushri P, Dzhagaryan A, Jovanov E, Milenkovic A (2016). A smartphone application suite for assessing mobility. Conf Proc IEEE Eng Med Biol Soc.

[31] Lathia N, Sandstrom G, Mascolo C, Rentfrow PJ (2017). Happier people live more active lives: using smartphones to link happiness and physical activity. PLoS One.

[32] Liu CT, Chan CT (2016). Exercise performance measurement with smartphone embedded sensor for well-being management. Int J Environ Res Public Health.

[33] Kelly D, Curran K, Caulfield B (2017). Automatic prediction of health status using smartphone-derived behavior profiles. IEEE J Biomed Health Inform.

[34] Juen J, Cheng Q, Schatz B (2015). A natural walking monitor for pulmonary patients using mobile phones. IEEE J Biomed Health Inform.

[35] Garcia-Ceja E, Osmani V, Mayora O (2016). Automatic stress detection in working environments from smartphones' accelerometer data: a first step. IEEE J Biomed Health Inform.

[36] Ben-Zeev D, Scherer EA, Wang R, Xie H, Campbell AT (2015). Next-generation psychiatric assessment: using smartphone sensors to monitor behavior and mental health. Psychiatr Rehabil J.

[37] Boonstra TW, Nicholas J, Wong QJ, Shaw F, Townsend S, Christensen H (2018). Using mobile phone sensor technology for mental health research: integrated analysis to identify hidden challenges and potential solutions. J Med Internet Res.

[38] Matthews M, Abdullah S, Murnane E, Voida S, Choudhury T, Gay G, Frank E (2016). Development and evaluation of a smartphone-based measure of social rhythms for bipolar disorder. Assessment.

[39] Beiwinkel T, Kindermann S, Maier A, Kerl C, Moock J, Barbian G, Rössler W (2016). Using smartphones to monitor bipolar disorder symptoms: a pilot study. JMIR Ment Health.

[40] Maxhuni A, Muñoz-Meléndez A, Osmani V, Perez H, Mayora O, Morales EF (2016). Classification of bipolar disorder episodes based on analysis of voice and motor activity of patients. Pervasive Mob Comput.

[41] Wang R, Aung MS, Abdullah S, Brian R, Campbell AT, Choudhury T, Hauser M, Kane J, Merrill M, Scherer AE (2016). Crosscheck: toward passive sensing detection of mental health changes in people with schizophrenia. Proceedings of the 2016 ACM International Joint Conference on Pervasive and Ubiquitous Computing.

[42] Saeb S, Lattie EG, Kording KP, Mohr DC (2017). Mobile phone detection of semantic location and its relationship to depression and anxiety. JMIR Mhealth Uhealth.

[43] Boukhechba M, Huang Y, Chow P, Fua K, Teachman BA, Barnes LE (2017). Monitoring social anxiety from mobilitycommunication patterns. Proceedings of the 2017 ACM International Joint Conference on Pervasive and Ubiquitous Computing and Proceedings of the 2017 ACM International Symposium on Wearable Computers.

[44] Ben-Zeev D, Wang R, Abdullah S, Brian R, Scherer AE, Mistler LA, Hauser M, Kane JM, Campbell A, Choudhury T (2016). Mobile behavioral sensing for outpatients and inpatients with schizophrenia. Psychiatr Serv.

[45] Buck B, Scherer E, Brian R, Wang R, Wang W, Campbell A, Choudhury T, Hauser M, Kane JM, Ben-Zeev D (2019). Relationships between smartphone social behavior and relapse in schizophrenia: a preliminary report. Schizophr Res.

[46] Farhan AA, Lu J, Bi J, Russell A, Wang B, Bamis A (2016). Multi-view bi-clustering to identify smartphone sensing features indicative of depression.

[47] Yue C, Ware S, Morillo R, Lu J, Shang C, Bi J, Kamath J, Russell A, Bamis A, Wang B (2018). Fusing location data for depression prediction. IEEE Trans Big Data.

[48] Boukhechba M, Daros AR, Fua K, Chow PI, Teachman BA, Barnes LE (2018). DemonicSalmon: monitoring mental health and social interactions of college students using smartphones. Smart Health.

[49] Pratap A, Atkins DC, Renn BN, Tanana MJ, Mooney SD, Anguera JA, Areán PA (2019). The accuracy of passive phone sensors in predicting daily mood. Depress Anxiety.

[50] Ben-Zeev D, Brian R, Wang R, Wang W, Campbell AT, Aung MS, Merrill M, Tseng VW, Choudhury T, Hauser M, Kane JM, Scherer EA (2017). CrossCheck: integrating self-report, behavioral sensing, and smartphone use to identify digital indicators of psychotic relapse. Psychiatr Rehabil J.

[51] Servia-Rodríguez S, Rachuri KK, Mascolo C, Rentfrow PJ, Lathi N, Sandstrom GM (2017). Mobile sensing at the service of mental well-being: a large-scale longitudinal study. Proceedings of the 26th International Conference on World Wide Web.

[52] Grünerbl A, Muaremi A, Osmani V, Bahle G, Ohler S, Tröster G, Mayora O, Haring C, Lukowicz P (2015). Smartphone-based recognition of states and state changes in bipolar disorder patients. IEEE J Biomed Health Inform.

[53] McNamara L, Ngai E (2018). SADHealth: a personal mobile sensing system for seasonal health monitoring. IEEE Syst J.

[54] Zhang H, Gashi S, Kimm H, Hanci E, Matthews O (2018). Moodbook: An application for continuous monitoring of social media usage and mood. Proceedings of the 2018 ACM International Joint Conference and 2018 International Symposium on Pervasive and Ubiquitous Computing and Wearable Computers.

[55] Jeong S, Breazeal CL (2016). Improving smartphone users' affect and wellbeing with personalized positive psychology interventions. Proceedings of the Fourth International Conference on Human Agent Interaction.

[56] Wang R, Campbell AT, Zhou X (2015). Using opportunistic face logging from smartphone to infer mental health: challenges and future directions. Adjunct Proceedings of the 2015 ACM International Joint Conference on Pervasive and Ubiquitous Computing and Proceedings of the 2015 ACM International Symposium on Wearable Computers.

[57] Shapsough S, Hesham A, Elkhorazaty Y, Zualkerman IA, Aloul F (2016). Emotion recognition using mobile phones.

[58] Triguero-Mas T, Donaire-Gonzalez D, Seto E, Valentín A, Martínez D, Smith G, Hurst G, Carrasco-Turigas G, Masterson D, van den Berg M, Ambròs A, Martínez-Íñiguez T, Dedele A, Ellis N, Grazulevicius T, Voorsmit M, Cirach M, Cirac-Claveras J, Swart W, Clasquin E, Ruijsbroek A, Maas J, Jerret M, Gražulevičienė R, Kruize H, Gidlow CJ, Nieuwenhuijsen MJ (2017). Natural outdoor environments and mental health: Stress as a possible mechanism. Environ Res.

[59] Saeb S, Zhang M, Karr CJ, Schueller SM, Corden ME, Kording KP, Mohr DC (2015). Mobile phone sensor correlates of depressive symptom severity in daily-life behavior: an exploratory study. J Med Internet Res.

[60] Wahle F, Kowatsch T, Fleisch E, Rufer M, Weidt S (2016). Mobile sensing and support for people with depression: a pilot trial in the wild. JMIR Mhealth Uhealth.

[61] Teles AS, Rocha A, da Silva E Silva FJ, Lopes JC, O'Sullivan D, van de Ven P, Endler M (2017). Enriching mental health mobile assessment and intervention with situation awareness. Sensors (Basel).

[62] Ng JKY, Wang J, Lam KL, Kam CH, Han S (2017). Capturing and analyzing pervasive data for smart health.

[63] Sahiti K, Kalanadhabhatta LM, Bhunia SS, Singhal A, Majethia R (2017). Smartphone-based qualitative analyses of social activities during family time. Proceedings of the First International Workshop on Human-centered Sensing, Networking, and Systems.

[64] Huang Y, Xiong H, Leach K, Zhang Y, Chow P, Fua K, Teachman BA, Barnes LE (2016). Assessing social anxiety using gps trajectoriespoint-of-interest data. Proceedings of the 2016 ACM International Joint Conference on Pervasive and Ubiquitous Computing.

[65] Luo L, Yang J, Bao X, Yan Z, Jiang Y (2015). SWAN: A novel mobile system to track and analyze social well-being. Adjunct Proceedings of the 2015 ACM International Joint Conference on Pervasive and Ubiquitous Computing and Proceedings of the 2015 ACM International Symposium on Wearable Computers.

[66] Rute S, Firdose S, Lopes LA, Moreira W, Mendes P (2016). NSense: A people centric, non-intrusive opportunistic sensing tool for contextualizing nearness.

[67] Singh VK, Goyal R, Wu S (2018). Riskalyzer: inferring individual risk-taking propensity using phone metadata. Proc ACM Interact Mob Wearable Ubiquitous Technol.

[68] Bati GF, Singh VK (2018). “Trust Us”: Mobile Phone Use Patterns Can Predict Individual Trust Propensity. Proceedings of the 2018 CHI Conference on Human Factors in Computing Systems.

[69] Pulekar G, Agu E (2016). Autonomously sensing loneliness and its interactions with personality traits using smartphones.

[70] Saeb S, Cybulski TR, Schueller SM, Kording KP, Mohr DC (2017). Scalable passive sleep monitoring using mobile phones: opportunities and obstacles. J Med Internet Res.

[71] Montanini L, Sabino N, Spinsante S, Gambio E (2018). Smartphone as unobtrusive sensor for real-time sleep recognition.

[72] Sarda A, Munuswamy S, Sarda S, Subramanian V (2019). Using passive smartphone sensing for improved risk stratification of patients with depression and diabetes: crosssectional observational study. JMIR Mhealth Uhealth.

[73] Lin HY, Wong BY, Lin SH, Chiu YC, Pan YC, Lee YH (2019). Development of a mobile application (App) to delineate "digital chronotype" and the effects of delayed chronotype by bedtime smartphone use. J Psychiatr Res.

[74] Nakano H, Hirayama K, Sadamitsu Y, Toshimitsu A, Fujita H, Shin S, Tanigawa T (2014). Monitoring sound to quantify snoring and sleep apnea severity using a smartphone: proof of concept. J Clin Sleep Med.

[75] Staples P, Torous J, Barnett I, Carlson K, Sandoval L, Keshavan M, Onnela JP (2017). A comparison of passive and active estimates of sleep in a cohort with schizophrenia. NPJ Schizophr.

[76] DeMasi O, Feygin S, Dembo A, Aguilera A, Recht B (2017). Well-being tracking via smartphone-measured activity and sleep: cohort study. JMIR Mhealth Uhealth.

[77] Lane ND, Lin M, Mohammod M, Yang X, Lu H, Cardone G, Ali S, Doryab A, Berke E, Campbell At, Choudhury T (2014). BeWell: sensing sleep, physical activities and social interactions to promote wellbeing. Mobile Netw Appl.

[78] Aslam H, Mukhtar H, Seemi F, Belaid D (2016). Harnessing smartphones as a personal informatics tool towards self-awareness and behavior improvement.

[79] Mo X, Shi D, Yang R, Li H, Tong ZH, Wang F (2015). A framework of fine-grained mobile sensing data collection and behavior analysis in an energy-configurable way.

[80] DeMasi O, Recht B (2017). A step towards quantifying when an algorithm cancannot predict an individual's wellbeing. Proceedings of the 2017 ACM International Joint Conference on Pervasive and Ubiquitous Computing and Proceedings of the 2017 ACM International Symposium on Wearable Computers.

[81] Siddiqui SA, Zhang Y, Feng Z, Kos A (2016). A pulse rate estimation algorithm using ppg and smartphone camera. J Med Syst.

[82] Huang RY, Dung LR (2016). Measurement of heart rate variability using off-the-shelf smart phones. Biomed Eng Online.

[83] García-Magariño I, Medrano C, Plaza I, Oliván B (2016). A smartphone-based system for detecting hand tremors in unconstrained environments. Pers Ubiquit Comput.

[84] Kostikis N, Hristu-Varsakelis D, Arnaoutoglou M, Kotsavasiloglou C (2015). A smartphone-based tool for assessing Parkinsonian hand tremor. IEEE J Biomed Health Inform.

[85] Lipsmeier F, Taylor KI, Kilchenmann T, Wolf D, Scotland A, Schjodt-Eriksen J, Cheng WY, Fernandez-Garcia I, Siebourg-Polster J, Jin L, Soto J, Verselis L, Boess F, Koller M, Grundman M, Monsch AU, Postuma RB, Ghosh A, Kremer T, Czech C, Gossens C, Lindemann M (2018). Evaluation of smartphone-based testing to generate exploratory outcome measures in a phase 1 Parkinson's disease clinical trial. Mov Disord.

[86] Liddle J, Ireland D, McBride SJ, Brauer SG, Hall LM, Ding H, Karunanithi M, Hodges PW, Theodoros D, Silburn PA, Chenery HJ (2014). Measuring the lifespace of people with Parkinson's disease using smartphones: proof of principle. JMIR Mhealth Uhealth.

[87] Wang R, Chen F, Chen Z, Li T, Harari G, Tignor S, Zhou X, Ben-Zeev D, Campbell AT (2014). StudentLife: assessing mental health, academic performance and behavioral trends of college students using smartphones. Proceedings of the 2014 ACM International Joint Conference on Pervasive and Ubiquitous Computing.

[88] Wang R, Harari G, Hao P, Zhou X, Cambpell AT (2015). Smartgpa: how smartphones can assess and predict academic performance of college students. Proceedings of the ACM International Joint Conference on Pervasive and Ubiquitous Computing.

[89] Baras K, Soares L, Paulo N, Barros R (2016). 'Smartphine': supporting students' well-being according to their calendar and mood.

[90] Tseng VW, Merrill M, Wittleder F, Abdullah S, Aung MH, Choudhury T (2016). Assessing mental health issues on college campuses: preliminary findings from a pilot study. Proceedings of the 2016 ACM International Joint Conference on Pervasive and Ubiquitous Computing: Adjunct.

[91] Hossain A, Poellabauer C (2016). Challenges in building continuous smartphone sensing applications.

[92] Vhaduri S, Munch A, Poellabauer C (2016). Assessing health trends of college students using smartphones.

[93] Harari GM, Gosling SD, Wang R, Chen F, Chen Z, Campbell AT (2017). Patterns of behavior change in students over an academic term: a preliminary study of activity and sociability behaviors using smartphone sensing methods. Comput Hum Behav.

[94] Chen F, Wang R, Zhou X, Campbell AT (2014). Department of Computer Science - Dartmouth College.

[95] Barbosa R, Nunes D, Figueira A, Aguiar H, Silva JS, Gonzalez F, Herrera C, Sinche S (2016). An architecture for emotional smartphones in Internet of Things.

[96] Vathsangam H, Sukhatme GS (2014). Using phone-based activity monitors to promote physical activity in older adults: a pilot study.

[97] Kelly D, Donnelly S, Caulfield B (2015). Smartphone derived movement profiles to detect changes in health status in COPD patients - a preliminary investigation. Conf Proc IEEE Eng Med Biol Soc.

[98] Nambi AU, Bannur S, Mehta I, Kalra H, Virmani A, Padmanabhan VN, Bhaskaran R, Bhandari R (2018). HAMS: Driver and Driving Monitoring using a Smartphone. Proceedings of the 24th Annual International Conference on Mobile Computing and Networking.

[99] Kau LJ, Chen CS (2015). A smart phone-based pocket fall accident detection, positioning, and rescue system. IEEE J Biomed Health Inform.

[100] Sie MR, Lo SC (2015). The design of a smartphone-based fall detection system.

[101] Vermeulen J, Willard S, Aguiar B, De Witte LP (2015). Validity of a smartphone-based fall detection application on different phones worn on a belt or in a trouser pocket. Assist Technol.

[102] Sansrimahachai W, Toahchoodee M, Piakaew R, Vijitphu T, Jeenboonmee S (2017). Real-time fall risk assessment system based on acceleration data.

[103] Chow KK, Leong BD, Lee BY (2018). Imagining consequences of excessive smartphone use via a character-based mobile application. Int J Ment Health Addiction.

[104] Fasoulis A, Virvou M, Tsihrintzis G, Patsakis C, Alepis E (2018). Sensus vox: Sentiment mapping through smartphone multi-sensory crowdsourcing.

[105] Bati GF, Singh VK (2017). Are you altruistic? Your mobile phone could tell.

[106] Wang R, Scherer EA, Walsh M, Wang W, Aung MH, Ben-Zeev D, Brian R, Campbell AT, Choudhury T, Hauser M, Kane J (2018). Predicting symptom trajectories of schizophrenia using mobile sensing. Proc ACM Interact Mob Wearable Ubiquitous Technol.

[107] Asensio-Cuesta S, Sánchez-García Á, Conejero JA, Saez C, Rivero-Rodriguez A, García-Gómez JM (2019). Smartphone sensors for monitoring cancer-related quality of life: app design, EORTC QLQ-C30 mapping and feasibility study in healthy subjects. Int J Environ Res Public Health.

[108] Ferdous R, Osmati V, Mayora O (2015). Smartphone app usage as a predictor of perceived stress levels at workplace.

[109] Rapeepisarn T, Tatiyanupanwong S, Kornvisitvatin B, Tangsripairoj S (2016). iRelief: an android application for smartphone syndrome prevention and treatment.

[110] Kanjo E, Kuss DJ, Ang CS (2017). NotiMind: utilizing responses to smart phone notifications as affective sensors. IEEE Access.

[111] Gu F, Kealy A, Khoshelham K, Shang J (2015). User-independent motion state recognition using smartphone sensors. Sensors (Basel).

[112] Weiss GM, Lockhart JW, Pulickal TT, McHugh T, Ronan IH, Timko JL (2016). Actitracker: a smartphone-based activity recognition system for improving health and well-being.

[113] Mukherjee A, Misra S, Mangrulkar P, Rajarajan M, Rahulamathavan Y (2017). SmartARM: a smartphone-based group activity recognition and monitoring scheme for military applications.

[114] Wu T, Wang L, Zheng Z, Wu S, Ma J, Tao X, Lu J (2018). Carmus: Towards a general framework for continuous activity recognition with missing values on smartphones.

[115] Canzian L, Musolesi M (2015). Trajectories of depression: unobtrusive monitoring of depressive states by means of smartphone mobility traces analysis. Proceedings of the 2015 ACM International Joint Conference on Pervasive and Ubiquitous Computing.

[116] Wan N, Lin G (2016). Classifying human activity patterns from smartphone collected GPS data: A fuzzy classification and aggregation approach. Trans GIS.

[117] Goodspeed R, Yan X, Hardy J, Vydiswaran VG, Berrocal VJ, Clarke P, Romero DM, Gomez-Lopez IN, Veinot T (2018). Comparing the data quality of global positioning system devices and mobile phones for assessing relationships between place, mobility, and health: field study. JMIR Mhealth Uhealth.

[118] Cheng Q, Juen J, Schatz BR (2014). Using mobile phones to simulate pulse oximeters: gait analysis predicts oxygen saturation. Proceedings of the 5th ACM Conference on Bioinformatics, Computational Biology, and Health Informatics.

[119] Azam MA, Shahzadi A, Khalid A, Anwar SM, Naeem U (2018). Smartphone based human breath analysis from respiratory sounds. Conf Proc IEEE Eng Med Biol Soc.

[120] Jannetts S, Schaeffler F, Beck J, Cowen S (2019). Assessing voice health using smartphones: bias and random error of acoustic voice parameters captured by different smartphone types. Int J Lang Commun Disord.

[121] Faurholt-Jepsen M, Bauer M, Kessing LV (2018). Smartphone-based objective monitoring in bipolar disorder: status and considerations. Int J Bipolar Disord.

[122] Rajagopalan A, Shah P, Zhang MW, Ho RC (2017). Digital platforms in the assessment and monitoring of patients with bipolar disorder. Brain Sci.

[123] Batra S, Baker RA, Wang T, Forma F, DiBiasi F, Peters-Strickland T (2017). Digital health technology for use in patients with serious mental illness: a systematic review of the literature. Med Devices (Auckl).

[124] Dogan E, Sander C, Wagner X, Hegerl U, Kohls E (2017). Smartphone based monitoring of objective and subjective data in affective disorders: where are we and where are we going? Systematic review. J Med Internet Res.

[125] Areàn PA, Hoa Ly K, Andersson G (2016). Mobile technology for mental health assessment. Dialogues Clin Neurosci.

[126] Þórarinsdóttir H, Kessing LV, Faurholt-Jepsen M (2017). Smartphone-based self- assessment of stress in healthy adult individuals: a systematic review. J Med Internet Res.

[127] Nguyen HN, Silva JN (2016). Use of smartphone technology in cardiology. Trends Cardiovasc Med.

[128] Ko PR, Kientz JA, Choe EY, Kay M, Landis CA, Watson NF (2015). Consumer sleep technologies: a review of the landscape. J Clin Sleep Med.

[129] Thomas JG, Bond DS (2014). Review of innovations in digital health technology to promote weight control. Curr Diab Rep.

[130] Kim BY, Lee J (2017). Smart devices for older adults managing chronic disease: a scoping review. JMIR Mhealth Uhealth.

[131] Cornet VP, Holden RJ (2018). Systematic review of smartphone-based passive sensing for health and wellbeing. J Biomed Inform.

[132] Harari GM, Lane ND, Wang R, Crosier BS, Campbell AT, Gosling SD (2016). Using smartphones to collect behavioral data in psychological science: practical considerations and challenges. Perspect Psychol Sci.

[133] Dennison L, Morrison L, Conway G, Yardley L (2013). Opportunities and challenges for smartphone applications in supporting health behavior change: qualitative study. J Med Internet Res.

[134] Torous J, Friedman R, Keshavan M (2014). Smartphone ownership and interest in mobile applications to monitor symptoms of mental health conditions. JMIR Mhealth Uhealth.

[135] Harari GM, Müller Sr, Mishra V, Wang R, Campbell AT, Rentfrow PJ, Gosling Sd (2017). An evaluation of students' interest in and compliance with self- tracking methods: recommendations for incentives based on three smartphone sensing studies. Soc Psychol Personal Sci.

[136] Anderson K, Burford O, Emmerton L (2016). Mobile health apps to facilitate self-care: a qualitative study of user experiences. PLoS One.

